# Supplementary material for: CMOS-based bio-image sensor spatially resolves neural activity-dependent proton dynamics in the living brain
Source: Nat Commun. 2020 Feb 5;11:712. doi: 10.1038/s41467-020-14571-y (PMC7002452; doi:10.1038/s41467-020-14571-y)
Supplement: Supplementary file 1 — Supplementary Information [file 41467_2020_14571_MOESM1_ESM.pdf]

CMOS-based bio-image sensor spatially resolves neural activity dependent proton dynamics in the living brain

Horiuchi *et al.*

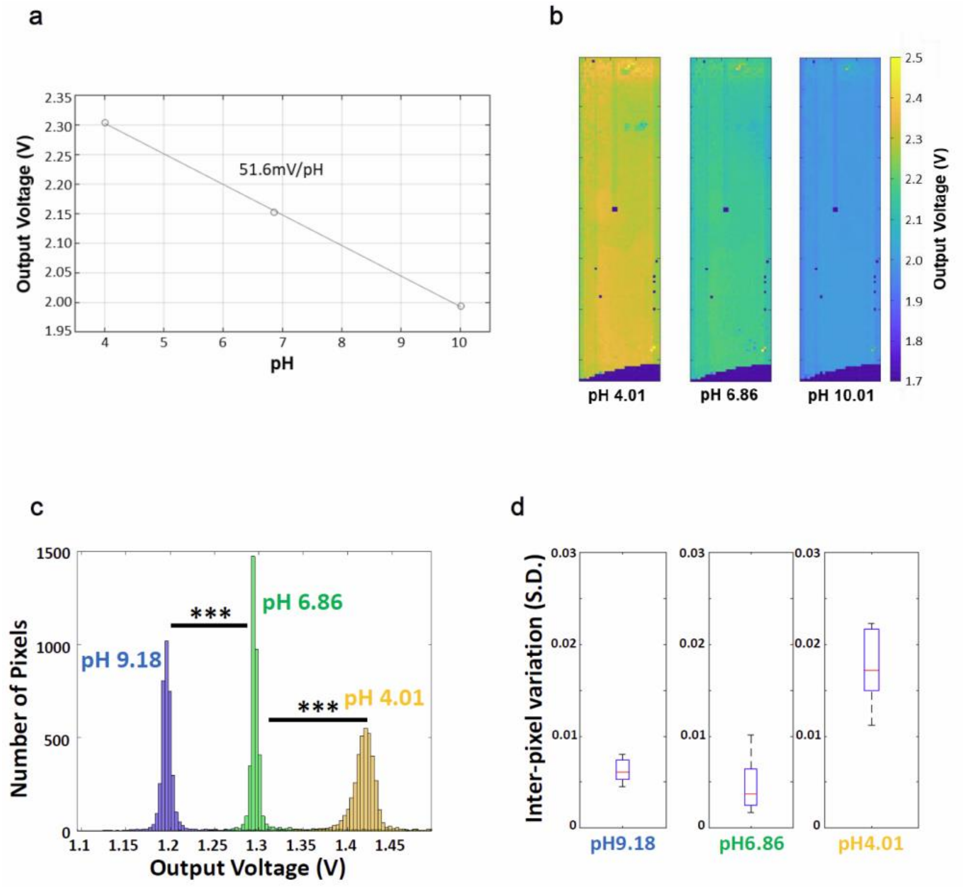

Supplementary Figure 1. pH sensitivity and pH response image of the pH sensor. **(a)** Representative voltage-pH standard curve. This was derived by immersing the proton sensing area into three pH calibration buffers which have a defined and stable pH (pH 4.01, 6.86, 10.01) and averaging the voltage output across all pixels. Linear regression was used to plot the relationship between solution pH and pixel voltage readout. The sensitivity of our sensor has been improved from 32.8 mV/pH to 51.6 mV/pH compared to previous 128 x 128 sensor [1]. **(b)** Representative voltage outputs of all pixels in a single proton image sensor when immersed into the three pH calibration buffers (pH 4.01, 6.86, 10.01). The dark pixels at the bottom mean defective pixels (average defective probability = 2.89%). **(c)** Histograms showing representative voltage outputs in the three different pH calibration buffers (pH 4.01, 6.86, 9.18). Distribution of individual pixels (n= 128 x 32 pixels) at each pH is shown in each color. A one-way ANOVA followed by Turkey's test indicated statistically significant difference in all combinations of groups (***, $p<0.001$). **(d)** SD of voltage outputs (i.e. inter-pixel variation) in the three pH calibration buffers (pH 4.01, 6.86, 9.18) summarized across 12 sensors. In the box and whisker plots, the center line indicates the median; the box defines the 25th - 75th percentiles; the whiskers represent min and max values. The source data underlying Supplementary Figure 1c and 1d are provided as a Source Data file.

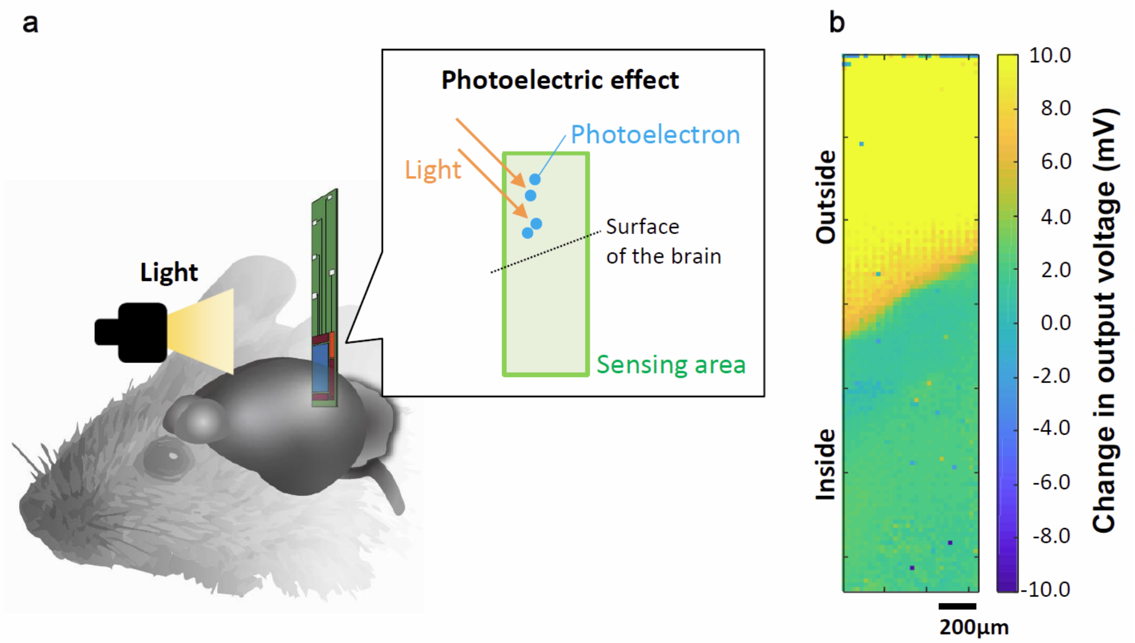

Supplementary Figure 2. Confirmation of insertion depth using photoelectric effect. **(a)** The pH sensing area of the proton image sensor was illuminated with white light following its insertion into the primary visual cortex (V1). Due to the photoelectric effect, the pixel voltage readout spikes dramatically when exposed to white light. This property enables the brain surface border to be defined. **(b)** The brain surface border was visualized based on the difference between voltage readouts during white light illumination and during the pre-exposure (dark) period.

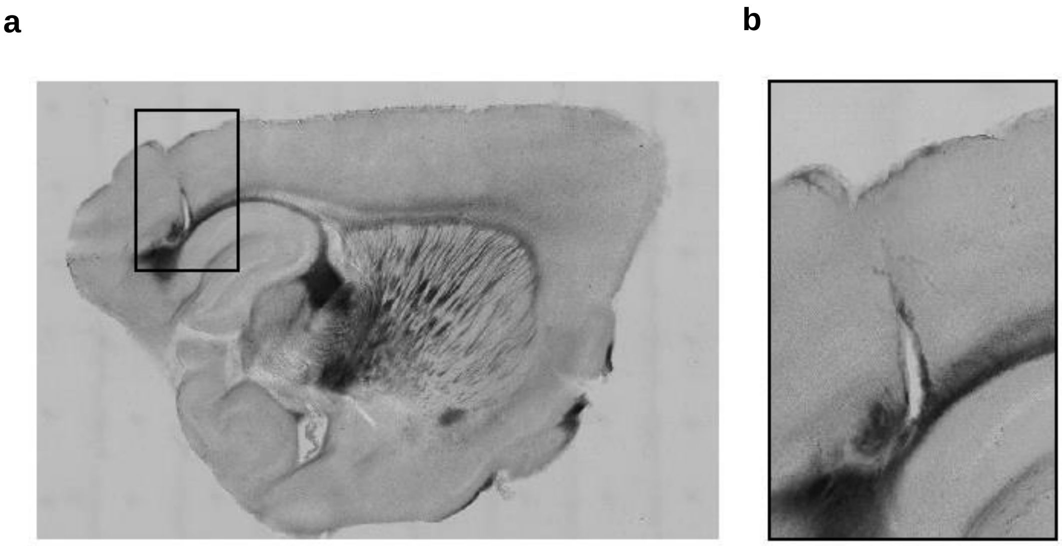

Supplementary Figure 3. Histology after the insertion of the pH sensor. (a) A sagittally sliced brain section prepared following proton image sensor insertion and completion of the pH measurement experiments. The black rectangle indicates the area that is magnified in (b). **(b)** A higher magnification view of the brain area containing the primary visual cortex (V1). The insertion tract of the proton image sensor can clearly be seen within the primary visual cortex which indicates that it was inserted correctly. The source data underlying Supplementary Figure 3a is provided as a Source Data file.

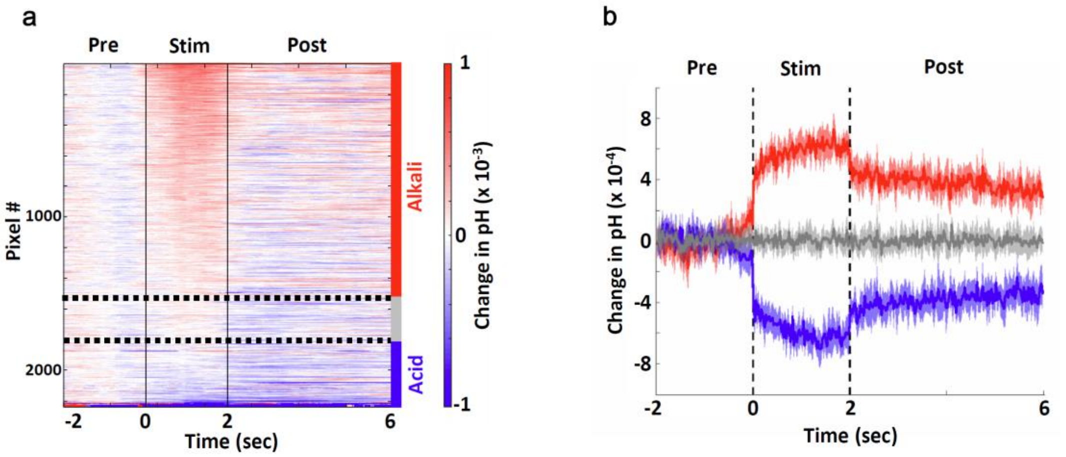

Supplementary Figure 4. Detection of temporal pH change in the brain during visual stimulation. (a) Peri-stimulus time histograms showing dynamic change in pH, at each pixel, triggered by the visual stimulation in a representative experiment. We calculated mean pH dynamics of each pixel before, during, and after the visual stimulation, over multiple trials. Pre: 2sec interval before each stimulation; Stim: 2sec stimulation; Post: 4sec interval after each stimulation. Time course change in pH averaged over 30 trials, at different pixels was shown (data from Fig. 2c or 5a, 90 degree, pixels in the brain region were used). Each averaged response pattern was categorized as alkaline response (red), neutral response (gray), or acidic response (blue) based on two-sample t-test ($p<0.05$). Data at each pixel was normalized by subtracting mean change in pH during pre-stim as baseline from the mean response over the time course. The data for different pixels were sorted according to the response categories and median values during visual stimulation. **(b)** Time course change of alkaline response (red), neutral response (gray), or acidic response (blue) summarized over all experiments. To calculate the temporal dynamics, the activation phase (starting from time zero) of response patterns were fitted using a two-component exponential equation (($\tau_{alkali,\ fast}$ = 250.2 msec, $\tau_{alkali,\ slow}$ = 14.19 sec, $\tau_{acid,\ fast}$ = 231.0 msec, $\tau_{acid,\ slow}$ = 6.99 sec)). Data for all pixels across 9 animals at all 8 directions are summarized. Each line and each shaded area indicate the median and the SD over animals (experiments) respectively. The source data underlying Supplementary Figure 4b is provided as a Source Data file.

Supplementary Reference

1. Futagawa, M. *et al.* Fabrication of a 128×128 pixels charge transfer type hydrogen ion image sensor. *IEEE Trans. Electron Devices* **60**, 2634-2639 (2013).
