# Supplementary material for: CMOS-based bio-image sensor spatially resolves neural activity-dependent proton dynamics in the living brain
Source: Nat Commun. 2020 Feb 5;11:712. doi: 10.1038/s41467-020-14571-y (PMC7002452; doi:10.1038/s41467-020-14571-y)
Supplement: Supplementary file 3 — Reporting Summary [file 41467_2020_14571_MOESM3_ESM.pdf]

nature research

Corresponding author(s): Junichi Nabekura, and Kazuaki Sawada

Last updated by author(s): Nov 30, 2019

Reporting Summary

Nature Research wishes to improve the reproducibility of the work that we publish. This form provides structure for consistency and transparency in reporting. For further information on Nature Research policies, see Authors & Referees and the Editorial Policy Checklist.

Statistics

For all statistical analyses, confirm that the following items are present in the figure legend, table legend, main text, or Methods section.

n/a	Confirmed	
☐	☒	The exact sample size (n) for each experimental group/condition, given as a discrete number and unit of measurement
☐	☒	A statement on whether measurements were taken from distinct samples or whether the same sample was measured repeatedly
☐	☒	The statistical test(s) used AND whether they are one- or two-sided<br>*Only common tests should be described solely by name; describe more complex techniques in the Methods section.*
☒	☐	A description of all covariates tested
☒	☐	A description of any assumptions or corrections, such as tests of normality and adjustment for multiple comparisons
☐	☒	A full description of the statistical parameters including central tendency (e.g. means) or other basic estimates (e.g. regression coefficient) AND variation (e.g. standard deviation) or associated estimates of uncertainty (e.g. confidence intervals)
☐	☒	For null hypothesis testing, the test statistic (e.g. F, t, r) with confidence intervals, effect sizes, degrees of freedom and P value noted<br>*Give P values as exact values whenever suitable.*
☒	☐	For Bayesian analysis, information on the choice of priors and Markov chain Monte Carlo settings
☒	☐	For hierarchical and complex designs, identification of the appropriate level for tests and full reporting of outcomes
☒	☐	Estimates of effect sizes (e.g. Cohen's d, Pearson's r), indicating how they were calculated

Our web collection on statistics for biologists contains articles on many of the points above.

Software and code

Policy information about availability of computer code

Data collection	The timing of the visual stimulus trials was controlled and recorded using a NI DAQ device (National Instruments Co. Ltd.) controlled by LabVIEW (version 2014; National Instruments Co. Ltd., Texas, USA) and custom written routines executed in MATLAB (R2017b; Math Works, Inc., Massachusetts, USA). All data were collected using custom-made software. After the proton imaging experiments, correct placement of the proton image sensor in the primary visual cortex (V1) was confirmed by viewing the brain slices using a Nikon A1R microscope (Nikon, Tokyo, Japan).
Data analysis	All data were analyzed using MATLAB (R2017b; Mathworks).

For manuscripts utilizing custom algorithms or software that are central to the research but not yet described in published literature, software must be made available to editors/reviewers. We strongly encourage code deposition in a community repository (e.g. GitHub). See the Nature Research guidelines for submitting code & software for further information.

Data

Policy information about availability of data

All manuscripts must include a data availability statement. This statement should provide the following information, where applicable:

- Accession codes, unique identifiers, or web links for publicly available datasets
- A list of figures that have associated raw data
- A description of any restrictions on data availability

The data that support the findings of this study are available from the corresponding authors upon reasonable request.

Field-specific reporting

Please select the one below that is the best fit for your research. If you are not sure, read the appropriate sections before making your selection.

☒ Life sciences ☐ Behavioural & social sciences ☐ Ecological, evolutionary & environmental sciences

For a reference copy of the document with all sections, see nature.com/documents/nr-reporting-summary-flat.pdf

Life sciences study design

All studies must disclose on these points even when the disclosure is negative.

Sample size	Sample size for each experiment is indicated in the figure legend for each experiment. The sample size was chosen based on previous experience for each experiment to yield high power to detect specific effects. No statistical methods were used to predetermine sample size.
Data exclusions	No data were excluded from the analyses.
Replication	All experimental findings were reliably reproducible.
Randomization	Each samples were randomly allocated into experimental groups.
Blinding	For all experiments, the investigators were blind to the experimental groups during the analyses.

Reporting for specific materials, systems and methods

We require information from authors about some types of materials, experimental systems and methods used in many studies. Here, indicate whether each material, system or method listed is relevant to your study. If you are not sure if a list item applies to your research, read the appropriate section before selecting a response.

Materials & experimental systems

n/a	Involved in the study	
☒	☐	Antibodies
☒	☐	Eukaryotic cell lines
☒	☐	Palaeontology
☐	☒	Animals and other organisms
☒	☐	Human research participants
☒	☐	Clinical data

Methods

n/a	Involved in the study	
☒	☐	ChIP-seq
☒	☐	Flow cytometry
☒	☐	MRI-based neuroimaging

Animals and other organisms

Policy information about studies involving animals; ARRIVE guidelines recommended for reporting animal research

Laboratory animals	Male, 8-10 week old, C57BL/6 mice housed under a 12 hr light/dark cycle with free access to food and water were used for all experiments.
Wild animals	This study did not involve wild animals.
Field-collected samples	This study did not involve samples collected from the field.
Ethics oversight	All animal experiments were approved by the National Institute for Physiological Sciences animal research committee.

Note that full information on the approval of the study protocol must also be provided in the manuscript.
